# Analysis of *Chromobacterium *sp. natural isolates from different Brazilian ecosystems

**DOI:** 10.1186/1471-2180-7-58

**Published:** 2007-06-21

**Authors:** Cláudia I Lima-Bittencourt, Spartaco Astolfi-Filho, Edmar Chartone-Souza, Fabrício R Santos, Andréa MA Nascimento

**Affiliations:** 1 Departamento de Biologia Geral, Instituto de Ciências Biológicas, Universidade Federal de Minas Gerais, Belo Horizonte, Minas Gerais, Brazil. Av. Antônio Carlos, 6627, CEP: 31.270-901, Brazil; 2 Universidade Federal do Amazonas, Manaus, Amazonas, Brazil

## Abstract

**Background:**

*Chromobacterium violaceum *is a free-living bacterium able to survive under diverse environmental conditions. In this study we evaluate the genetic and physiological diversity of *Chromobacterium *sp. isolates from three Brazilian ecosystems: Brazilian Savannah (Cerrado), Atlantic Rain Forest and Amazon Rain Forest. We have analyzed the diversity with molecular approaches (16S rRNA gene sequences and amplified ribosomal DNA restriction analysis) and phenotypic surveys of antibiotic resistance and biochemistry profiles.

**Results:**

In general, the clusters based on physiological profiles included isolates from two or more geographical locations indicating that they are not restricted to a single ecosystem. The isolates from Brazilian Savannah presented greater physiologic diversity and their biochemical profile was the most variable of all groupings. The isolates recovered from Amazon and Atlantic Rain Forests presented the most similar biochemical characteristics to the *Chromobacterium violaceum *ATCC 12472 strain. Clusters based on biochemical profiles were congruent with clusters obtained by the 16S rRNA gene tree. According to the phylogenetic analyses, isolates from the Amazon Rain Forest and Savannah displayed a closer relationship to the *Chromobacterium violaceum *ATCC 12472. Furthermore, 16S rRNA gene tree revealed a good correlation between phylogenetic clustering and geographic origin.

**Conclusion:**

The physiological analyses clearly demonstrate the high biochemical versatility found in the *C. violaceum *genome and molecular methods allowed to detect the intra and inter-population diversity of isolates from three Brazilian ecosystems.

## Background

*Chromobacterium violaceum *is a Gram-negative bacterium found in the environment as a saprophyte, in a wide variety of tropical and subtropical ecosystems, primarily in water and soil [1]. It is a β-Proteobacterium that is of great biotechnological interest due to its wide potential for industrial, pharmacological and ecological use [2].

This free-living bacterium presents a high flexibility to survive in the most diverse environments [3]. Its biological characteristics make *C. violaceum *a major component of the microbiota in tropical ecosystems. In Brazil, *C. violaceum *is present in three main ecosystems: the Amazon Rain Forest (AmF) [4], the Brazilian Savannah (BS), also called Cerrado, and the Atlantic Rain Forest (AtF), which are considered biodiversity hotspots [5]. These three ecosystems encompass altogether almost 50% of the total area in the Neotropical region.

The complete genome of *C. violaceum *strain ATCC 12472 confirmed its considerable potential for several biotechnological applications [6]. However, it should be pointed out that the genome was sequenced from a laboratory strain, which does not necessarily reflect the diversity of natural isolates of the same species. Besides, the sequenced strain ATCC 12472 was isolated from soil in Malaysia, and it has been maintained in the laboratory for many years. Therefore, the aims of this study are focused in the evaluation of the genetic and physiological diversity of *C. violaceum *isolated from three Brazilian ecosystems. In addition, we performed phylogenetic analyses of the isolates along with other members of the *Neisseriaceae *family by using 16S rRNA gene sequences and amplified ribosomal DNA restriction analysis (ARDRA). We have also compared the phylogenetic trees with the phenogram based on the antimicrobial resistance and biochemical tests of the isolates.

## Results

### Phenotypic characterization

Forty three isolates (26, 11 and 6 from Brazilian Savannah, Amazon and Atlantic Rain Forests, respectively) were analyzed in this study. None of the isolates was able to grow at 4°C and all grew at 15°C,25°C and 37°C. Although in early stages all isolates showed violet pigmentation, either on solid or liquid medium, the color intensity was variable. In addition, after several subcultures, some isolates stopped presenting the typical pigmentation.

**Figure 1:**
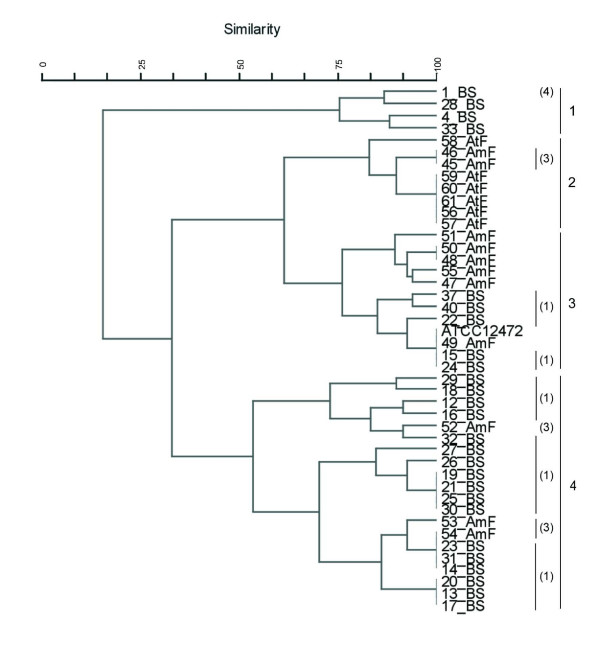
**Cluster analysis of *Chromobacterium *sp. isolates and of *C. violaceum  *ATCC 12472 according to API 20E profiles**. A distance matrix of simple similarity coefficients was clustered with the UPGMA algorithm.

Data from API 20E and additional tests are summarized in Table 1 and Fig. 1. The API 20E system failed to identify any isolate including the ATCC 12472 strain as being *C. violaceum*. The isolates recovered from Amazon and Atlantic Rain Forests were the most similar to the ATCC 12472 strain characteristics (Table 1). The ATCC 12472 strain fermented neither glucose nor sucrose, and only 9% of isolates from Amazon Rain Forest fermented the two substrates simultaneously. On the other hand, all the isolates from Atlantic Rain Forest fermented glucose and none fermented sucrose. In addition, no isolate from Atlantic and Amazon Rain Forests used citrate as carbon source, in accordance with Bergey's manual of systematic bacteriology [7]. The isolates from Brazilian Savannah presented greater physiologic diversity. Only two out of 22 biochemical tests performed (H_2_S and TDA) did not produce a reaction in the Brazilian Savannah's isolates.

**Table 1:** Phenotypic characteristics of *Chromobacterium *sp. isolates.

Biochemical Characteristics	Percentage of positive bacterial isolates			
	
		Geographic Regions		
		
	Type strain	ERF (11)*	AF (6)	BS (26)
β-galactosidase (ONPG)	-	0	0	19
Arginine dihydrolase (ADH)	+	91	100	50
Lysine decarboxylase (LDC)	-	0	0	15
Ornithine decarboxylase (ODC)	-	0	0	12
Citrate (CIT)	-	0	0	46
H_2_S production (H_2_S)	-	0	0	0
Urease (URE)	-	0	0	19
Tryptophanane deaminase (TDA)	-	0	0	0
Indole production (IND)	-	64	100	8
Acetoin production (VP)	-	36	100	69
Gelatinase (GEL)	+	100	83	73
Fermentation/oxidation:				
Glucose (GLU)	-	9	100	19
Mannitol (MAN)	-	0	0	19
Inositol (INO)	-	0	0	4
Sorbitol (SOR)	-	0	0	19
Rhamnose (RHA)	-	0	0	8
Sucrose (SAC)	-	9	0	12
Melibiose (MEL)	-	0	0	15
Amygdalin (AMY)	-	0	0	15
Arabinose (ARA)	-	0	0	8
Motility (MOT)	+	100	83	81
MacConkey (McC)	+	82	83	85

* number of isolates; + positive; - negative.

The phenogram derived from biochemical profiles data is shown in Fig. 1. Four main clusters were found. Cluster 1 comprised four isolates from Brazilian Savannah, and its biochemical profile was the most dissimilar of all groupings. Cluster 2 consisted of eight isolates from Atlantic and Amazon Rain Forests. In this clustering analysis, the isolates from Amazon Rain Forest showed the same biochemical profile and five isolates from Atlantic Rain Forest also shared a common biochemical profile. Cluster 3 included 11 isolates from Amazon Rain Forest and Brazilian Savannah and also the ATCC 12472 strain. Three isolates presented the same biochemical profile as the ATCC 12472 strain. The third and largest cluster was formed by 20 isolates from Amazon Rain Forest and Brazilian Savannah, the majority of isolates was coming from the later ecosystem.

The degree of resistance in the three populations of the isolates is given by MIC for 50% (MIC50) and 90% (MIC90) of isolates (Table 2). Analysis of MIC revealed that, as expected, there was a wide range in the inhibitory concentration to a particular antimicrobial agent as well as among the populations. As expected, β-lactam-resistant isolates were predominant. The isolates 12BS and 59AtF were the only ones to be inhibited by < 2 μg/ml of ampicillin. In order to analyze β-lactamase production, a colorimetric assay was performed in the isolates resistant to ampicillin. We found that all isolates were β-lactamase producers.

**Table 2:** Minimum inhibitory concentration which 50% and 90% of *Chromobacterium *sp. isolates in the population overall are inhibited (μgml^-1^).

		Origin						
		
			BS		AF		ERF	
			
Antimicrobials	Range	Type strain	MIC50	MIC90	MIC50	MIC90	MIC50	MIC90
Ap	2–1024	1024	> 1024	> 1024	512	> 1024	1024	> 1024
Am	2–1024	256	1024	> 1024	16	256	256	1024
Cf	2–128	> 128	> 128	> 128	≤ 2	≤ 2	> 128	> 128
Ak	2–128	< 2	16	64	16	16	16	16
Gm	2–128	< 2	4	64	4	8	4	16
Km	2–128	4	16	64	32	32	8	16
Sm	2–128	16	32	128	16	16	32	64
Cm	2–128	32	32	128	4	16	16	128
Rf	2–128	32	16	128	≤ 2	≤ 2	16	32
Nx	2–128	< 2	4	16	8	8	≤ 2	32
Tc	2–128	< 2	≤ 2	4	≤ 2	≤ 2	≤ 2	32
Hg	2–16	8	≤ 2	8	≤ 2	≤ 2	≤ 2	4

**Figure 2:**
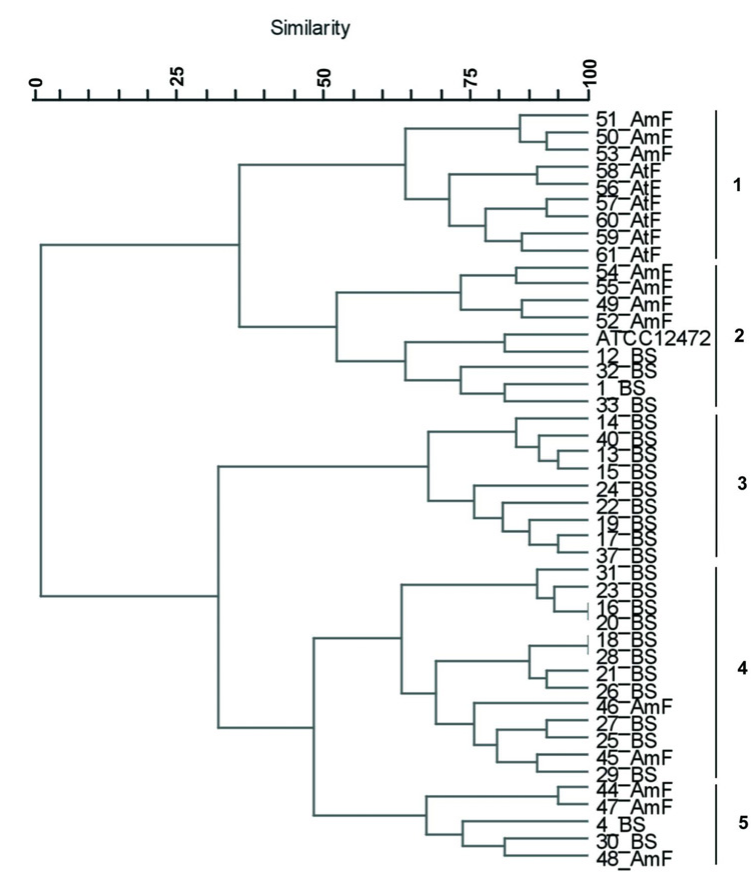
**Cluster analysis of *Chromobacterium *sp. isolates and of *C. violaceum *ATCC 12472 according to antimicrobial susceptibility profiles**. A distance matrix of simple similarity coefficients was clustered with the UPGMA algorithm.

A phenogram based on the MIC profiles revealed that almost all isolates exhibited a distinct profile for a combination of the used antibiotics. However, some isolates presented identical patterns (Fig. 2). The main clusters were defined with a cut off similarity of about 50%. Cluster 3 was exclusively formed by isolates from Brazilian Savannah. Clusters 1, 2, 4 and 5 grouped isolates from the three ecosystems whereas the type strain was included in cluster 2. Cluster 4, the largest group formed by 13 isolates, mainly from Brazilian Savannah with two pairs of isolates showing identical MIC profiles.

### 16S rRNA gene analysis

**Figure 3:**
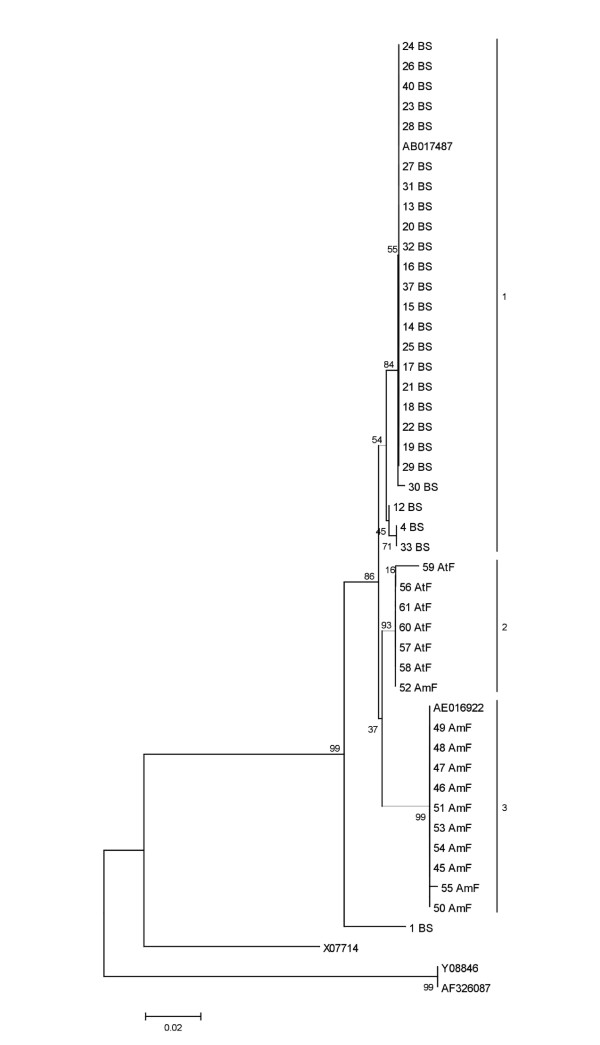
**Phylogenetic tree based on 16S rDNA partial sequences of *Chromobacterium *sp. isolates and of strains used as references, including *C. violaceum *ATCC 12472**. One thousand bootstrap resamplings were used to evaluate robustness of the inferred trees. AE016922, *C. violaceum *ATCC 12472; AB017487, *Chromabacterium *sp. MBIC3901; X07714, *Neisseria gonorrhoeae *and Y08846 and AF326087,*  Janthinobacterium lividum*.

**Figure 4:**
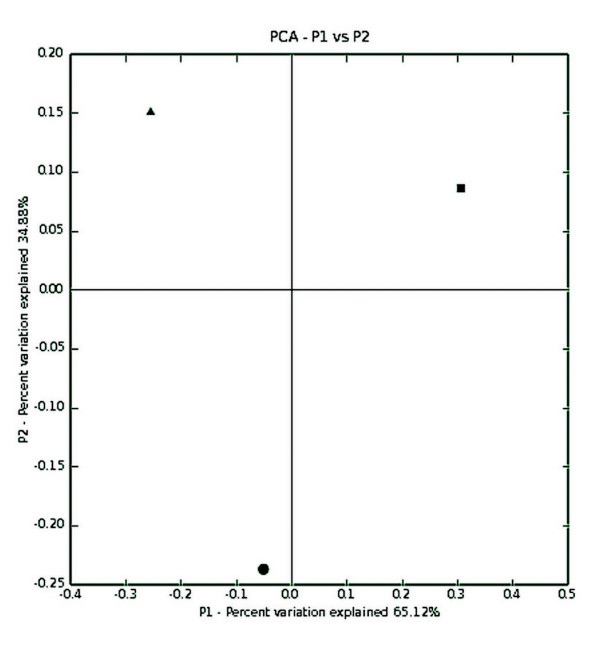
**Principal components analysis ordination plot for the 16S rRNA gene**. The percent of variation explained by each principal component is indicated on the axis labels. Ecosystems are represented by the following symbols: AmF ■, AtF ●, and BS ▲.

The sequences analyzed in this study ranged from positions 99 to 483 of the 16S rRNA gene. The phylogenetic tree showed that isolates usually clustered according to their geographic origin. The only exception was the Amazon isolate 52ERF, which grouped with Atlantic Rain Forest isolates (Fig. 3). In order to compare the association between genetic similarity and specific features of the ecosystems, we used the UniFrac metric analysis. This analysis revealed three main clusters of related isolates that match the geographic origin. The robustness of the inferred UniFrac tree topology to the presence of specific isolates represented was confirmed by jackknife analysis (*P *< 0.001). Principal components analyses also suggested that there are significant differences among ecosystems (*P *< 0.001, Fig. 4). The average similarity of 16S rRNA gene sequences between the type strain and the isolates was of 98.5%. The highest degree of similarity observed was between type strain and Amazon Rain Forest isolates (99.6%). Indeed, nine out of eleven Amazon Rain Forest isolates shared identical 16S rRNA gene sequences with the type strain. The lowest degree of average similarity observed was between the type strain and Atlantic Rain Forest isolates with a value of 99.1%, and an individual from Brazilian Savanah (1BS – Fig. 3) presented the highest divergence. According to the phylogenetic analysis, isolates from Amazon Rain Forest and Brazilian Savannah seemed to have a closer relationship with the type strain than isolates from Atlantic Rain Forest.

### ARDRA analysis

**Figure 5:**
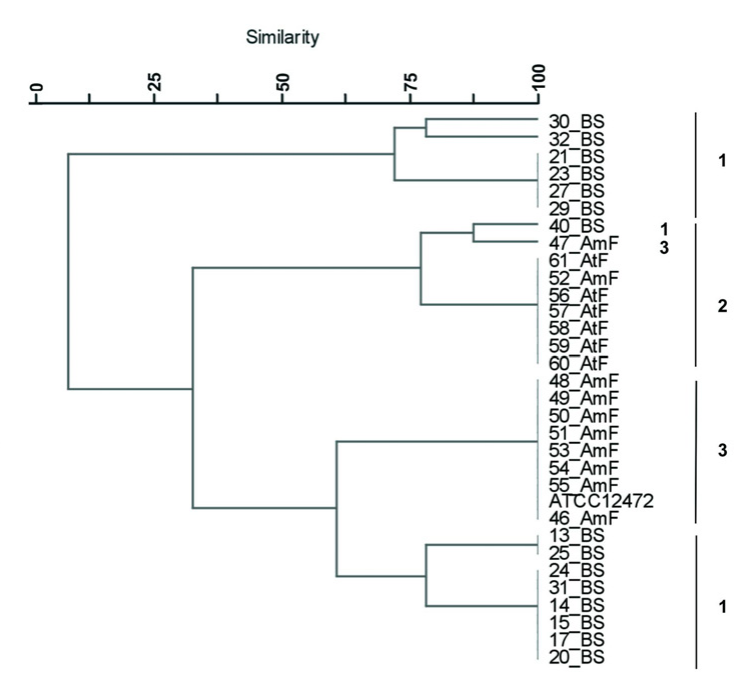
**Cluster analysis of *Chromobacterium *sp. isolates and of *C. violaceum *ATCC 12472 according ARDRA profiles**. A distance matrix of simple similarity coefficients was clustered with the UPGMA algorithm. Numbers 1 to 3 identify the 16S rDNA sequence based phylogeny clusters obtained with the *Chromobacterium *sp. isolates.

The complete 16S rRNA gene amplicon was digested separately with three restriction enzymes. Each endonuclease generated three to five profiles: *Bfa*I (three profiles),*  Afl*III (four profiles) and *Nla*IV (five profiles). In this study, ARDRA profiles were obtained for 31 isolates and four main clusters were identified (Fig. 5). Brazilian Savannah isolates were grouped in two separate clusters that were previously identified as cluster 1 in the 16S rRNA sequence tree (Fig. 2). Cluster 2 assembled all isolates from Atlantic Rain Forest, found in 16S rRNA gene cluster, plus 40BS and 47AmF belonging to clusters 1 and 3, respectively, of 16S rRNA gene tree. Cluster 3 presented a similar grouping as presented by the 16S rRNA gene sequence phylogeny (Fig. 3).

## Discussion

The isolates in this study used more different substrates than the type strain. In agreement with the specifications of the API 20E kit for identification of the *C. violaceum *species, 99% of the strains express the enzyme arginine dihydrolase, and they are gelatinase positive, glucose fermenters, mobile, and grow in MacConkey agar. Seventy five percent of the strains use citrate as a source of carbon. Only 14% produce indol, and 10% ferment sucrose. However, Holt and Krieg [6], in Bergey's Manual of Systematic Bacteriology, require other positive tests to consider a microorganism as *C. violaceum*. For instance, 60% of the described strains ferment sorbitol and 50% ferment rhamanose, whereas the API 20E testing kit manual affirms that no strain use those two substrates.

It is also important to consider that environmental isolates can modify their physiological characteristics because of nutrients availability. In addition, changes in gene expression can occur to reduce the energy expenses [8]. Thus, the physiological variation found in the isolates in this study can be explained by the differences in the nutrient supply of this environment causing changes in phenotype expression or acquisition of inherited adaptive characteristics by horizontal gene transfer or selective pressure. Furthermore, the similar physiological characteristics found in the isolates from the Amazon and Atlantic Rain Forests can be related to the slightly resemblance of the two environments. Both are forests with high precipitation rate and comparable ecological characteristics.

*C. violaceum *is a free-living bacterium which can rarely become an opportunist pathogen infecting humans. Antimicrobial susceptibility data usually are obtained from clinical cases [9]. After the genome sequencing, comparative genomic analyses revealed a large number of antibiotic resistance genes. Among the 57 genes found, the most important ones were those related to β-lactam and multidrug resistance [10]. In the present study, we observed a great variety of susceptibility profiles in the environmental isolates. As expected, the isolates were more resistant to β-lactam antibiotics. However, the resistance to aminoglycosides was also high, but no resistance genes for these antibiotics were identified in *C. violaceum *genome so far. Again, the isolates from Brazilian Savannah were distinguished from the other ecosystems as they presented higher values of MIC90 for ten antibiotics and for mercury. The only exception was the resistance for tetracycline, which was higher in Amazon Rain Forest isolates. In contrast, the isolates from Atlantic Rain Forest were more divergent, presenting lower MIC90 values.

Although the 16S rRNA gene is not usually suitable for analysis of intraspecific diversity, the chosen region presents the most heterogeneous part of the entire gene [11]. The data obtained herein demonstrated that this method allowed grouping the *Chromobacterium *sp. isolates according to geographical regions. In contrast, other bacteria (*Escherichia coli*, *Salmonella enterica*, *Bacillus cereus *and *B. anthracis*) present lower 16S rRNA genetic diversity, particularly considering the single cluster observed in *B. cereus* and *B. anthracis *(100% similarity, data not shown). These data are interesting since the *E. coli *complete genomes [12] reveal a large genomic variability as length and gene content, although the genetic diversity in 16S rRNA genes is not as high in the *E. coli *sequenced genomes, as in *Chromobacterium *sp. Therefore, for *Chromobacterium *sp. isolates we could expect the same or more genome variability due to its apparently high genetic and phenotypic diversity. In addition, the physiological methods revealed similar genetic diversity to 16S rRNA data. Clusters based on biochemical profiles were congruent with clusters obtained by the 16S rRNA gene tree.

The biochemical phenogram and the phylogenetic tree indicated a high genetic and phenotypic diversity of the Brazilian Savannah isolates, which were quite distinct from the reference strain. The ARDRA method demonstrated to be useful for intraspecific analysis. This method revealed a remarkable diversity of Brazilian Savannah isolates which formed two clusters, while these isolates were identical in the 16S rRNA gene sequence analysis. On the other hand, Atlantic Rain Forest isolates demonstrated lower genetic diversity as illustrated by ARDRA, biochemical and MIC profiles. Interestingly, these isolates demonstrated to be more susceptible to aminoglycosides. It should be pointed out that one of the resistance mechanisms to aminoglycosides relies on mutations in the 16S rRNA gene, which could be related to the lower genetic diversity found in the isolates from Atlantic Rain Forest.

## Conclusion

The physiological analyses clearly demonstrate the high biochemical versatility found in *C. violaceum *genome. Besides, the molecular methods revealed the genetic diversity found within and between populations from three Brazilian ecosystems investigated.

## Methods

### Study area

Serra do Cipó National Park (Brazilian Savannah or Cerrado) and Rio Doce State Park (Atlantic Rain Forest) are located in the Minas Gerais State. Brazilian Savannah presents vegetation composed mainly by grasses and bushes, and the sampled river is located in high altitude fields (> 1,200 m). The Atlantic Rain Forest site consists of a State reserve that includes around 50 lagoons surrounded by primary and secondary forests. The Negro River, the third sampling site, is a large tributary (1,750 Km) of the Amazon basin that presents dark transparent water, located in the Amazon Rain Forest.

### Water sampling

The water samples were collected in sterilized glass bottles and stored on ice for until six hours, before subsequent procedures in the laboratory. Each sample was collected at a depth of approximately 15–20 cm from the surface.

### Bacterial isolation and reference strain

Aliquots of 0.1 ml of sampled water were inoculated without dilution in Petri dishes containing 1/4 nutrient agar (NA, Difco Laboratories) and incubated at 25°C up to seven days. Bacterial isolates used for further studies were purified from single violet colonies. Following, isolates were incubated at 4°C, 15°C and 37°C on 1/4 NA [13]. *C. violaceum *ATCC 12472 was used as reference strain in all analyses.

Biochemical and susceptibility testing

API20E (BioMérieux, Marcy l'Etoile, France) testing was performed following the manufacturer's instructions. The results were interpreted with the Analytical Profile Index (API) database of the ApiLab Plus software (version 3.3.3; BioMérieux, Marcy l'Etoile, France). Other tests were performed to detect motility using Motility Test Medium (Difco Laboratories) and ability to grow in MacConkey Agar (Difco Laboratories). The minimum inhibition concentration (MIC) was determined by the agar dilution method performed in Mueller-Hinton medium (MH; Difco Laboratories). Antimicrobial susceptibilities to ampicillin (Ap), amoxicillin-clavulanic acid (Am), tetracycline (Tc), chloramphenicol (Cm), nalidixic acid (Nx), rifampicin (Rf), amikacin (Ak), gentamicin (Gm), kanamycin (Km), streptomycin (Sm) cefotaxime (Cf) and the heavy metal – mercury bichloride (Hg) were tested. All antimicrobials were obtained from Sigma Chemical Co. and mercury was obtained from Merck Co.

### Detection of β-lactamase production

Beta-lactamase activity was tested with nitrocefin (Calbiochem, San Diego, Calif., USA) as described by Braga et. al [14].

### Clustering analysis of phenotypical tests

For cluster analysis, the data were converted into a binary matrix, where the digit 1 represents the presence of a phenotypic character, and the digit 0 its absence. The similarity matrix was generated by Euclidean distances, which were used to build a tree with the unweighted pair group mean averages (UPGMA) algorithm. Analysis of phenotypic data was performed using the software PAST [15].

### 16S ribosomal RNA gene amplification

The complete 16S rRNA gene was amplified by PCR using the primers PA [16] and U2 [17]. Polymerase chain reaction mixtures (20 μl) consisted of 0.4 mM of each dNTP, 0.5 μM of each primer, 1 unit of *Taq *DNA polymerase (Phoneutria, Brazil), and 40 ng of bacterial DNA. The thermal cycling conditions consisted in one cycle at 95°C for 10 min followed by 30 cycles of 30 s of denaturation at 95°C, 40 s of annealing at 48°C, and 2 min of extension at 72°C, and a final extension step of 15 min at 72°C.

### Amplified ribosomal RNA restriction analysis (ARDRA)

The amplicons were digested separately with *Bfa*I, *Afl*II and *Nla*IV (New England BioLabs Inc.), according to the supplier's instructions. *Bfa*I,*  Afl*II and *Nla*IV were previously selected using the NEBcutter V2.0 software (New England BioLabs Inc.). Restriction fragments were resolved by 8% polyacrylamide gel electrophoresis and the band patterns were compared in order to define operational taxonomic units (OTUs).

### 16S ribosomal RNA gene sequence analysis

The 16S rRNA gene partial sequencing was made utilizing the primers PA and CFV1 (5' -TTAACGCTYGCACCCTACG- 3'). Sequencing reactions were performed by using standard protocols with DYEnamic ET dye terminator kit (Amersham Biosciences) and the MegaBACE 1000 capillary sequencer (Amersham Biosciences). Each sequence in forward and reverse directions was repeated at least three times for every bacterial isolate. The 16S rRNA gene sequences were basecalled, checked for quality, aligned and analyzed using Phred v.0.20425 [18], Phrap v.0.990319 [19] and Consed 12.0 [20] software. Phylogenetic analysis was inferred by MEGA 3 software [21] using the neighbor-joining method [22] calculated by the Kimura method [23]. One thousand bootstrap resamplings were used to evaluate robustness of the inferred trees. Additional 16S rRNA gene sequences of *C. violaceum *(AE016922 and AB017487), *Neisseria gonorrhoeae *(X07714) and *Janthinobacterium lividum *(Y08846 and AF326087) were obtained from GenBank Database. *N. gonorrhoeae *and *J. lividum *were used as outgroups. UniFrac [24] was used to test for statistical differences between isolates from distinct ecosystems. First, a phylogenetic tree was built for the 16S rRNA gene sequences using the neighbor-joining method as implemented in MEGA 3. Second, a test was carried out to detect differences between isolates from distinct ecosystems and collecting times, using the UniFrac statistics software that performed a principal components analyses.

### Nucleotide sequence accession number

The individual 16S rRNA gene sequences were deposited in the GenBank Data Library under accession numbers EF077669–EF077711.

## Authors' contributions

CIL-B carried out laboratory work and wrote the draft of manuscript. SAF was responsible for the *Chromobacterium *sp. samples from the Amazon Rain Forest. FRS and ECS helped to conceive the design of the study and to write the final manuscript, as well as the sampling in the Savannah together CIL-B. AMAN conceived the design of the study, coordinated the project, and helped to write the final manuscript. All authors have read and approved the final manuscript.
